# Analysis of Individuals from a Dengue-Endemic Region Helps Define the Footprint and Repertoire of Antibodies Targeting Dengue Virus 3 Type-Specific Epitopes

**DOI:** 10.1128/mBio.01205-17

**Published:** 2017-09-19

**Authors:** Daniela V. Andrade, Leah C. Katzelnick, Doug G. Widman, Angel Balmaseda, Aravinda M. de Silva, Ralph S. Baric, Eva Harris

**Affiliations:** a Division of Infectious Diseases and Vaccinology, School of Public Health, University of California, Berkeley, Berkeley, California, USA; b Department of Epidemiology, Gillings School of Global Public Health, University of North Carolina at Chapel Hill, Chapel Hill, North Carolina, USA; c Laboratorio Nacional de Virología, Centro Nacional de Diagnóstico y Referencia, Ministerio de Salud, Managua, Nicaragua; d Department of Microbiology and Immunology, University of North Carolina School of Medicine, Chapel Hill, North Carolina, USA; Johns Hopkins Bloomberg School of Public Health

**Keywords:** dengue virus, natural infection, neutralizing antibodies, quaternary epitope, repertoire, serotype-specific

## Abstract

The four dengue virus serotypes (DENV1 to 4) cause dengue, a major public health problem worldwide. Individuals exposed to primary DENV infections develop serotype-specific neutralizing antibodies, including strongly neutralizing antibodies targeting quaternary epitopes. To date, no studies have measured the levels and kinetics of serum antibodies directed to such epitopes among populations in regions where dengue is endemic. Here, we use a recombinant DENV4 (rDENV4/3-M14) displaying a major DENV3 type-specific quaternary epitope recognized by human monoclonal antibody 5J7 to measure the proportion, magnitude, and kinetics of DENV3 type-specific neutralizing antibody responses targeting this epitope. Primary DENV3 sera from 30 individuals in a dengue hospital-based study in Nicaragua were studied 3, 6, 12, and 18 months post-infection, alongside samples collected annually 1 to 4 years post-primary DENV3 infection from 10 individuals in a cohort study in Nicaragua. We found substantial individual variation in the proportion of DENV3 type-specific neutralizing antibody titers attributed to the 5J7 epitope (range, 0 to 100%), with the mean significantly increasing from 22.6% to 41.4% from 3 to 18 months. We extended the transplanted DENV3 5J7 epitope on the virion (rDENV4/3-M16), resulting in increased recognition in several individuals, helping define the footprint of the epitope. However, 37% and 13% of the subjects still showed little to no recognition of the 5J7 epitope at 3 and 18 months, respectively, indicating that one or more additional DENV3 type-specific epitopes exist. Overall, this study demonstrates how DENV-immune plasma from populations from areas of endemicity, when coupled with structurally guided recombinant viruses, can help characterize the epitope-specific neutralizing antibody response in natural DENV infections, with direct implications for design and evaluation of dengue vaccines.

## IMPORTANCE

The four serotypes of dengue virus cause dengue, a major public health burden worldwide, yet it has been challenging to develop a vaccine that is safe and equally effective against all four serotypes. More in-depth characterization of natural human neutralizing antibody responses is needed to identify determinants of protective antibody responses to all DENV serotypes. Here, we use hospital and cohort studies in a region where dengue is endemic to assess the proportion and kinetics of the DENV3 neutralizing antibody response directed to a quaternary epitope on DENV3 recognized by strongly neutralizing human monoclonal antibody 5J7, which was transplanted into a DENV4 backbone. We show that many individuals recognized the 5J7 epitope, but to various degrees over time, suggesting that additional DENV3-specific epitopes likely exist. Thus, characterization of epitope-specific neutralizing antibody responses in natural DENV infections can help define the footprint and repertoire of antibodies directed to DENV3 type-specific epitopes, with implications for dengue vaccine development.

## INTRODUCTION

Dengue is the most prevalent mosquito-borne viral disease in humans, caused by infection with four antigenically distinct serotypes of dengue virus (DENV1 to 4), an enveloped, positive-sense RNA flavivirus. A range of clinical manifestations can occur during DENV infection, from undifferentiated illness and classic dengue fever (DF) to the most severe forms, dengue hemorrhagic fever/dengue shock syndrome (DHF/DSS), characterized by plasma leakage, shock, and potentially death (1). One of the hallmarks of dengue is the fact that sequential infections with different serotypes can be either protective or pathogenic. While antibodies elicited by primary infection usually confer lifelong protection against the homologous serotype, cross-reactive neutralizing responses can wane over time. In the event of a secondary DENV infection, it is thought that the risk of severe disease may be increased via pre-existing cross-reactive weakly neutralizing or non-neutralizing antibodies that facilitate virus entry into target Fcγ receptor-bearing cells (2–5). Given this risk factor, it is imperative that vaccines induce protective immunity to all 4 serotypes simultaneously.

The DENV envelope (E) protein is the major target of neutralizing antibodies (6), and its ectodomain is comprised of three domains: EDI, EDII, and EDIII. E facilitates virus attachment to host cells and subsequent fusion with the endosomal membrane. Recent findings have shown that potently neutralizing human monoclonal antibodies (hMAbs) are directed to complex quaternary epitopes present only on the E protein as assembled on the dengue virion, not as the recombinant monomer (recE) (7–10). Within this class of highly neutralizing hMAbs is 5J7, which was isolated from a donor with a history of primary DENV3 infection (7). Cryo-electron microscopy (cryo-EM) studies of the 5J7 hMAb showed that a single Fab molecule binds three distinct E proteins on the surface of the virion—the EDI/EDII hinge region of one E monomer and domains II and III of two adjacent E proteins—although it is less clear whether contact points in all three monomers are essential for neutralization potency (9). Subsequent studies employed a reverse-genetics approach to transplant the core of the quaternary epitope targeted by 5J7 into a DENV1 backbone to create an rDENV1/3 chimeric virus, which was partially sensitive to neutralization by the hMAb 5J7 (11). Importantly, when administered prophylactically, 5J7 was shown to reduce the viral load in a mouse model infected with DENV3 or rDENV1/3 (11). Based on the characterization of the DENV2 type-specific MAb 2D22, a different chimera, rDENV4/2, was created by transplanting the DENV2 EDIII onto a DENV4 backbone (12). The DENV2-specific antibody response in polyclonal sera of individuals who experienced a natural primary DENV2 infection or were immunized with a monovalent live attenuated DENV2 vaccine tracked with the DENV2 type-specific quaternary structure epitope(s) on the rDENV4/2 virus (12). These observations demonstrate the utility of chimeric DENV viruses as tools for tracking epitope-specific neutralizing antibody responses in immune sera following DENV infection or vaccination.

In the present study, we used two recombinant DENV4 strains with expanded transplants of the 5J7 quaternary epitope to measure the level and kinetics of neutralizing antibodies directed to this epitope following natural primary DENV3 infection in Nicaragua (13) up to 4 years postinfection. Our results show that the 5J7 epitope is either moderately or highly targeted by DENV3 type-specific neutralizing antibodies by 63 and 87% of the individuals analyzed at 3 and 18 months, respectively, and the proportion of the DENV3 neutralizing response attributable to the partial 5J7 epitope increases over time. Our results also suggest that the DENV3 antibody repertoire contains other type-specific neutralizing epitope(s) yet to be characterized. Our analysis of the recognition of a quaternary DENV epitope in a population living in a region where dengue is endemic serves as a model for measuring the fine specificity of antibodies to DENV after natural infection or vaccination.

## RESULTS

### Study participants.

Thirty patients who were enrolled for suspected dengue between 2010 and 2011 at the Nicaraguan national pediatric reference hospital, Hospital Infantil Manual de Jesús Rivera (HIJMR), were selected for analysis of plasma collected 3, 6, 12, and 18 months post-infection. All patients were laboratory confirmed as DENV3 positive by reverse transcription-PCR (RT-PCR) and/or virus isolation. All experienced a primary DENV infection, with 20/30 (67%) manifesting disease as DF and 10/30 (33%) classified as DHF. In addition, 10 individuals participating in a long-term pediatric cohort study who experienced a primary DENV3 infection between 2009 and 2010 and manifested disease as DF were chosen to analyze plasma samples 1 to 4 years post-illness.

### Longitudinal analysis of DENV-specific neutralizing antibodies following primary DENV3 infection.

**FIG 1:**
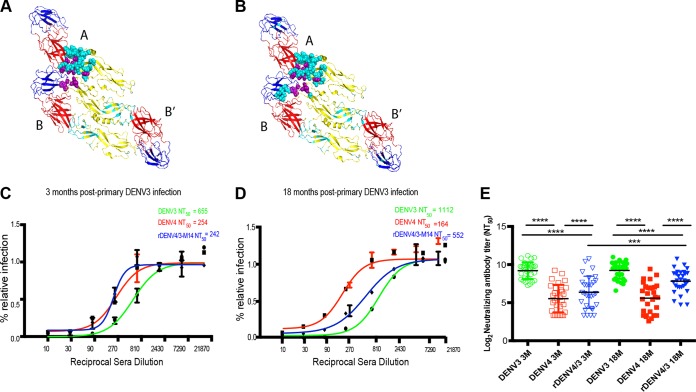
Neutralizing antibody titers to the chimeric rDENV4/3-M14 virus and parental DENV3 and DENV4 viruses 3 and 18 months post-primary DENV3 infection. (A) Ribbon diagram of the DENV E trimer (domains I, II, and III in red, yellow, and blue, respectively), showing amino acid residues within the 5J7 epitope spanning the A, B, and B′ monomers (purple and teal spheres); the residues represented by the teal spheres were transplanted into a DENV4 backbone, creating the rDENV4/3-M14 virus. (B) As in panel A, but the residues represented by the teal spheres were transplanted into a DENV4 backbone to create the rDENV4/3-M16 virus. (C and D) The raw antibody titration data were fitted with a four-parameter sigmoidal dose-response curve to estimate the 50% neutralizing antibody titer (NT_50_) to the rDENV4/3-M14 and parental viruses DENV3 and DENV4 in plasma samples 3 and 18 months postinfection, respectively. (E) Geometric mean of the NT_50_ values to the rDENV4/3-M14 and parental viruses of DENV3 and DENV4 in plasma samples 3 and 18 months postinfection. Data are representative of two independent experiments processed in duplicate for each plasma sample. The NT_50_ values were compared by two-way ANOVA (*n =* 30). ***, *P* < 0.001; ****, *P* < 0.0001.

Following primary DENV infection, a durable serotype-specific neutralizing response can be detected years after exposure (14). To determine whether the 5J7 epitope is recognized by the DENV3 type-specific antibody response in a setting where dengue is endemic, neutralization assays were performed with primary DENV3 plasma collected 3 and 18 months post-infection and the rDENV4/3-M14 virus displaying the partial 5J7 epitope (D. Widman, E. Young, U. Nivarthi, S. R. Royal, J. Swanstrom, L. Y. Boyd, K. Debbink, M. Begley, A. M. de Silva, W. B. Messer, and R. Baric, unpublished) (Fig. 1A). Sigmoidal dose-response curves representative of one primary DENV3 infection show neutralization of the rDENV4/3-M14 virus at 3 and 18 months post-infection at levels close to parental DENV3 at the later time point (Fig. 1C and D). As expected, in all subjects, primary DENV3 plasma strongly neutralized DENV3, with titers significantly higher than those to DENV4 virus (Fig. 1E). The neutralizing titers to the parental strain UNC 3001 and the infecting DENV3 Nicaraguan strain were similar at both time points (see Fig. S1 in the supplemental material), as both are members of DENV3 genotype III (15). Plasma from most individuals neutralized the rDENV4/3-M14 virus, and some neutralized it at levels similar to DENV3 (Fig. 1E). This indicates that DENV3 type-specific neutralizing antibodies in human DENV3-immune plasma recognized the 5J7 amino acid residues transplanted into the DENV4 backbone. The longitudinal analysis showed a significant increase in the neutralizing titers to the rDENV4/3-M14 at 18 months (*P* < 0.001), while the neutralizing titers to the parental viruses did not show significant variation between the time points analyzed (Fig. 1E).

10.1128/mBio.01205-17.1 FIG S1  Neutralizing antibody titers of primary DENV3 plasma against the parental DENV3 UNC 3001 strain and the DENV3 Nicaraguan strain. Neutralization assays were performed in parallel with the UNC 3001 and Nicaraguan DENV3 strains circulating during the 2010-2011 epidemics for plasma samples collected 3 and 18 months post-primary DENV3 infection (*n =* 2). Download FIG S1, PDF file, 0.1 MB. Copyright © 2017 Andrade et al. 2017 Andrade et al. This content is distributed under the terms of the Creative Commons Attribution 4.0 International license.

### Correlation between neutralizing antibody titers to rDENV4/3-M14 and DENV3 at 3 and 18 months post-primary DENV3 infection.

**FIG 2:**
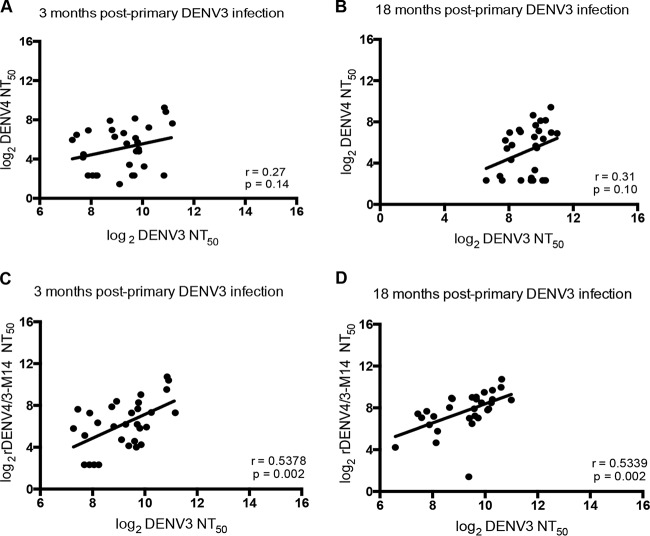
Correlation analysis between DENV3 and DENV4 or rDENV4/3-M14 neutralizing antibody titers over time. (A and B) Correlation between DENV3 NT_50_ and DENV4 NT_50_ at 3 and 18 months following primary DENV3 infection, respectively. (C and D) Correlation between DENV3 NT_50_ and rDENV4/3-M14 NT_50_ at 3 and 18 months following primary DENV3 infection, respectively. Data are representative of two independent experiments performed in duplicate for each plasma sample (*n =* 30). Pearson correlation coefficients (*r*) were calculated between the pairs of NT_50_ values. Linear regression lines are shown.

Following primary infection, a type-specific response to the infecting serotype and a cross-reactive response to heterologous DENV serotypes is developed (7). In individuals who experienced a primary DENV3 infection, the correlation between the DENV3 50% neutralizing antibody titer (NT_50_) and the DENV4 cross-reactive NT_50_ in plasma was not significant at 3 and 18 months post-infection (Fig. 2A and B). To investigate how the rDENV4/3-M14 virus neutralizing antibody titers track with the DENV3 response, correlation analyses between rDENV4/3-M14 and DENV3 NT_50_ values were performed at 3 and 18 months post-infection. At both time points analyzed, this correlation was significant (Fig. 2C and D). Thus, the increased positive correlation between DENV3 and rDENV4/3-M14, compared to the correlation between DENV3 and DENV4, is due to inclusion of the 5J7 epitope transplant in the DENV4 backbone.

### Proportion and kinetics of the DENV3 type-specific neutralizing response attributable to the 5J7 epitope.

**FIG 3:**
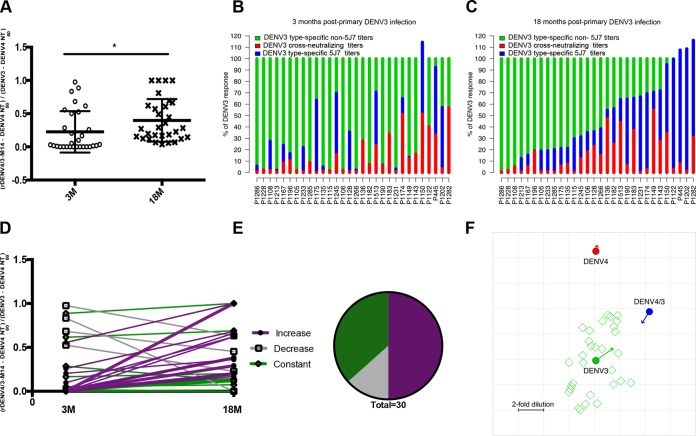
Proportion and kinetics of the DENV3 type-specific neutralizing antibody response attributable to the 5J7 epitope. (A) The proportion of the DENV3 type-specific response directed to the 5J7 epitope obtained with 3- and 18-month post-infection samples. Results for 3 and 18 months (*n =* 30) were compared by paired *t* test, where * represents *P* < 0.05. (B and C) Proportion of the rDENV4/3-M14 and DENV4 neutralizing antibody response relative to DENV3 NT_50_, where the *y* axis represents 100% of the DENV3 NT_50_ values at 3 and 18 months post-infection, respectively. Individual codes are ordered by the proportion of DENV3 attributable to rDENV4/3-M14 observed at 18 months. (D) Individual trajectories of the proportion of the DENV3 type-specific response directed to the 5J7 epitope at 3 and 18 months (*n =* 30). (E) Pie chart representing the percentage of individuals who displayed a constant, decreased, or increased proportion of the rDENV4/3-M14 DENV3 response between 3 and 18 months post-primary DENV3 infection (*n =* 30). (F) Antigenic cartography map generated from NT_50_. The antigenic cartography map positions viruses (closed circles [rDENV4/3-M14, DENV3, and DENV4]) and plasma (open squares) as points, with the distance between each virus and plasma derived from its respective neutralization titer (see Materials and Methods). Each grid square corresponds to a 2-fold dilution in the neutralization titer. Each open square represents one of the 30 plasma samples analyzed.

The proportion of the DENV3 type-specific response attributable to the 5J7 epitope recognition was measured as (rDENV4/3-M14 NT_50_ − DENV4 NT_50_)/(DENV3 NT_50_ − DENV4 NT_50_), the ratio between rDENV4/3-M14 titers and DENV3 NT_50_ from which the cross-reactive component (DENV4 NT_50_) had been subtracted. The mean proportion of DENV3 type-specific response targeted to the 5J7 epitope was 22.6% at 3 months post-infection, and at 18 months post-infection, the proportion significantly increased to 41.4% (*P* = 0.0184) (Fig. 3A). The proportions were not significantly different between individuals who experienced DF versus DHF (see Fig. S2 in the supplemental material).

10.1128/mBio.01205-17.2 FIG S2  Proportion of the DENV3 neutralizing antibody response attributable to the 5J7 epitope in individuals with different disease severity classifications. The analysis of the proportion of the DENV3 neutralizing antibody response attributable to the 5J7 epitope in individuals who experienced DF (*n =* 20) and DHF (*n =* 10) at 3 and 18 months post-infection was tested for significance with a two-way ANOVA and was found to be nonsignificant. Download FIG S2, PDF file, 0.04 MB. Copyright © 2017 Andrade et al. 2017 Andrade et al. This content is distributed under the terms of the Creative Commons Attribution 4.0 International license.

To visualize the proportions of the DENV3 neutralizing antibody response attributable to cross-reactive neutralization (DENV4 NT_50_) or the 5J7 epitope (rDENV4/3-M14 NT_50_ not explained by DENV4 NT_50_), we plotted the ratios of DENV4 NT_50_ to DENV3 NT_50_ and rDENV4/3-M14 NT_50_ to DENV3 NT_50_, where the percentage of the DENV3 neutralizing antibody response is represented on the *y* axis (Fig. 3B and C). The 30 individuals are aligned in an ascending order of proportion of the DENV3 response explained by rDENV4/3-M14 at 18 months. It is evident that, at both 3 and 18 months, there was extensive variability in the amount to which the DENV3 response is explained by 5J7 or cross-reactive neutralization. Further, there was variation by individual in changes in neutralizing antibody responses over time, with most individuals having an increasing proportion of the DENV3 response attributable to the 5J7 epitope, while several lost 5J7 recognition (Fig. 3C).

As the prevalence of this type-specific epitope among individuals living in regions where dengue is endemic is an important guide for vaccine design, the individual variation of the proportion was further analyzed. The proportion of the DENV3 neutralizing antibody response directed to the 5J7 epitope ranged from 0 to 97% at 3 months and from 6 to 100% at 18 months post-infection across the 30 individuals studied (Fig. 3A to C). The proportions of the DENV3 neutralizing response attributable to the 5J7 epitope were defined as low (0 to 10%), moderate (10 to 30%), and high (above 30%). At 3 months, 57% (17/30) showed inexistent to low recognition of the 5J7 epitope, 13% (4/30) displayed moderate recognition, and 30% (9/30) displayed high recognition of the 5J7 epitope. At 18 months, 13% (4/30), 47% (14/30), and 40% (12/30) of the individuals displayed low, moderate, and high recognition of the 5J7 epitope, respectively. Matched-pair analysis enabled assessment of the change of the proportion in each individual over time (Fig. 3D). Using 1 standard deviation of the average of the proportions at 3 and 18 months as the criterion to define change, 50% (15/30) of the individuals displayed an increase in the proportion over time, while 37% (11/30) showed no alteration and 13% (4/30) had a decreased proportion (Fig. 3E).

Next, we analyzed the data using antigenic cartography, where the NT_50_ was treated as a “distance” between antisera and rDENV4/3-M14 as well as its parental viruses. A convergent pattern over time was observed, with rDENV4/3-M14 and DENV3 moving toward each other at 18 months post-primary DENV3 infection and DENV4 virus staying about the same distance from the primary DENV3 anti-sera (Fig. 3F). This indicates greater antigenic similarity between DENV3 and rDENV4/3-M14 as recognized by the primary infection antisera at the later time point, with the antibody response becoming increasingly specific to the 5J7 epitope over time.

### Trajectories of the rDENV4/3-M14, DENV3, and cross-reactive neutralizing responses.

**FIG 4:**
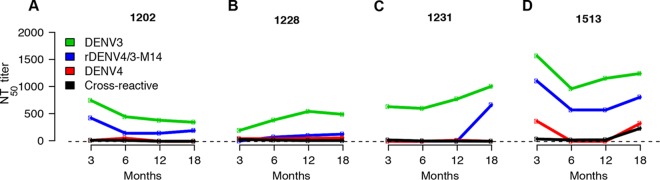
Trajectories of neutralizing antibody responses 3, 6, 12, and 18 months post-primary DENV3 infection. The neutralizing antibody titers to rDENV4/3-M14, DENV3, DENV4, and the overall cross-reactive titers (geometric mean of DENV1, DENV2, and DENV4 NT_50_ values) from 12 individuals were analyzed. (A) rDENV4/3-M14 titers follow similar trajectory to DENV3 titers in an individual with a constant proportion of the 5J7 response. (B) Increase of rDENV4/3-M14 titers between 3 and 6 months, with parallel decay of cross-reactive titers, indicates affinity maturation in an individual who displayed an increased proportion of the 5J7 response. (C) Increase in rDENV4/3-M14 titers between 12 and 18 months indicates possible homotypic reexposure in an individual with an increased proportion of the 5J7 response. (D) Increase in cross-reactive titers between 12 and 18 months indicates possible heterotypic reexposure in an individual who displayed a decreased proportion of the 5J7 response over time.

While the cross-reactive neutralizing antibody response has been described to wane over time (7), recent studies in settings where dengue is endemic have described the maintenance of cross-reactive titers, potentially due to “boosting” by homotypic or heterotypic reinfection that fell short of the antibody threshold for a new infection (16, 17). To investigate whether the constant, increased, or decreased proportion of the neutralizing antibody titers targeting the 5J7 epitope between 3 and 18 months is associated with the change in magnitude of homologous and heterologous titers, samples collected at 6 and 12 months from a subset of individuals (*n =* 12) were used for neutralization assays to rDENV4/3-M14 and all DENV serotypes (DENV1 to 4) (Fig. 4; see Fig. S3 in the supplemental material). In this analysis, in addition to DENV4, the cross-reactive titers as averages of anti-DENV1, 2, and 4 titers were plotted. As an example of a constant proportion between 3 and 18 months, one subject (Fig. 4A) displayed rDENV4/3-M14 titers that followed similar kinetics to the DENV3 NT_50_ at 3, 6, 12, and 18 months, while the cross-reactive titers decayed over time. This pattern was observed in 42% (5/12) of the individuals analyzed. As an illustration of an increased proportion, subject 1228 (Fig. 4B) showed an increase in DENV3 and rDENV4/3-M14 titers between 3 and 6 months in parallel with a slight decrease of cross-reactive titers; one case (8%) with this pattern was seen. Another example of increased titers from 3 to 18 months is shown with subject 1231 (Fig. 4C), where the increase of DENV3 and rDENV4/3-M14 titers is observed at the later time points—between 12 and 18 months. This pattern, observed in 33% (4/12) of the subjects, suggests a possible homotypic boost, whereby reinfection with DENV3 may have occurred (18), as the effect is only seen in the homologous titers (Fig. 4C). Finally, the pattern of decreased proportion is observed in subject 1513 (Fig. 4D), where an increase in neutralizing titers to the heterologous serotypes is seen at the later time points—between 12 and 18 months—which could indicate heterotypic boosting. This pattern was observed in 17% (2/12) of the individuals analyzed.

10.1128/mBio.01205-17.3 FIG S3  Trajectories of neutralizing antibody responses at 3, 6, 12, and 18 months of a subset of 8 individuals in the hospital study. In the top panel, each line represents the NT_50_ for each time point analyzed (3, 6, 12, and 18 months) for DENV3 (green), rDENV4/3-M14 (blue), DENV4 (red), or the mean of cross-reactive titers (DENV1, DENV2, and DENV4) (black). (A) Trajectory of rDENV4/3-M14 and DENV3 neutralizing antibody titers between 3 and 18 months indicates affinity maturation in the early time points post-infection. (B to D) Increase of rDENV4/3-M14 at the later time points (6 or 12 months postinfection) indicates possible homotypic boosting. (E to H) Increase of cross-reactive neutralizing antibody titers at the later time points post-infection indicates possible heterotypic reexposure. Download FIG S3, PDF file, 0.04 MB. Copyright © 2017 Andrade et al. 2017 Andrade et al. This content is distributed under the terms of the Creative Commons Attribution 4.0 International license.

### Proportion of the DENV3 neutralizing response targeted to the 5J7 epitope years after infection.

**FIG 5:**
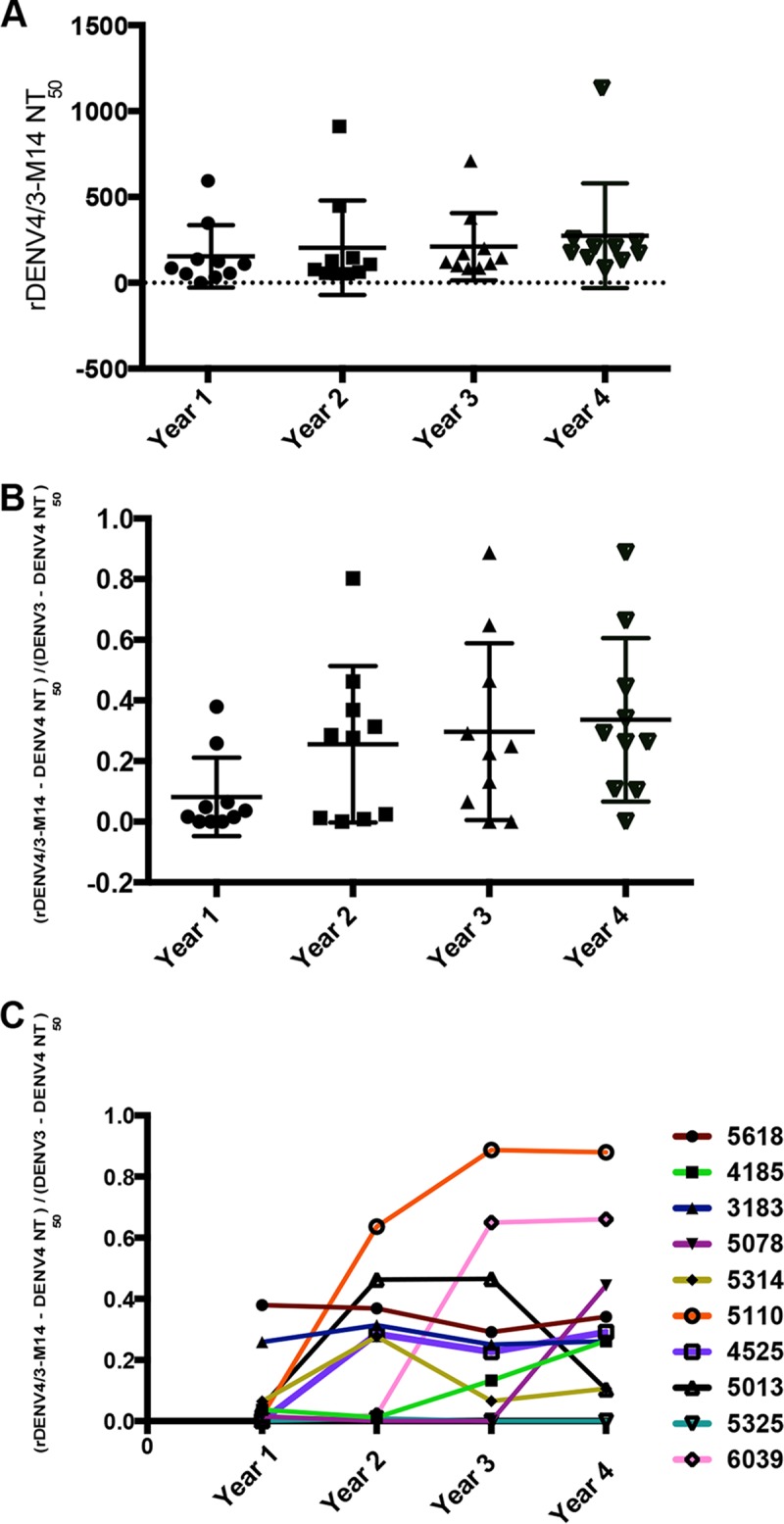
Proportion of the DENV3 type-specific neutralizing response targeted to the 5J7 epitope up to 4 years after infection in a cohort study design. (A) Neutralizing titers to the rDENV4/3-M14 virus from year 1 through year 4 post-primary DENV3 infection in a cohort study setting. (B) The ratio of the 5J7 epitope neutralizing antibody response (rDENV4/3-M14) to that of the parental DENV3 was analyzed from 1 to 4 years after primary DENV3 infection. (C) Individual trajectories of the DENV4/3-M14/DENV3 proportions from year 1 through year 4 post-primary DENV3 infection. Data are representative of two independent experiments processed in duplicate for each plasma sample. The NT_50_ and the proportion of the 5J7 response were compared by one-way ANOVA (*n =* 10); no significant differences were observed.

To test whether the durability of the DENV3 neutralizing antibody response targeted to the 5J7 epitope is maintained beyond 18 months post-infection, primary DENV3 plasma samples collected up to 4 years after infection in a long-term cohort study were analyzed. While the rDENV4/3-M14 neutralizing titers did not change across the years (Fig. 5A), the proportion trended toward an increase between year 1 and year 2 after infection (Fig. 5B). From year 1 through year 4, the mean proportions were 8, 25, 29, and 34%, respectively, across the 10 individuals analyzed (Fig. 5C). The individual trajectories of the proportion indicate variations of 0 to 0.26, 0 to 0.80, 0 to 0.87, and 0 to 0.89 at years 1 to 4 (Fig. 5C). The long-term analysis of the titers to the rDENV4/3-M14 virus indicates that the response to the 5J7 epitope is stable and durable.

### rDENV4 virus containing a larger footprint of the 5J7 epitope region.

**FIG 6:**
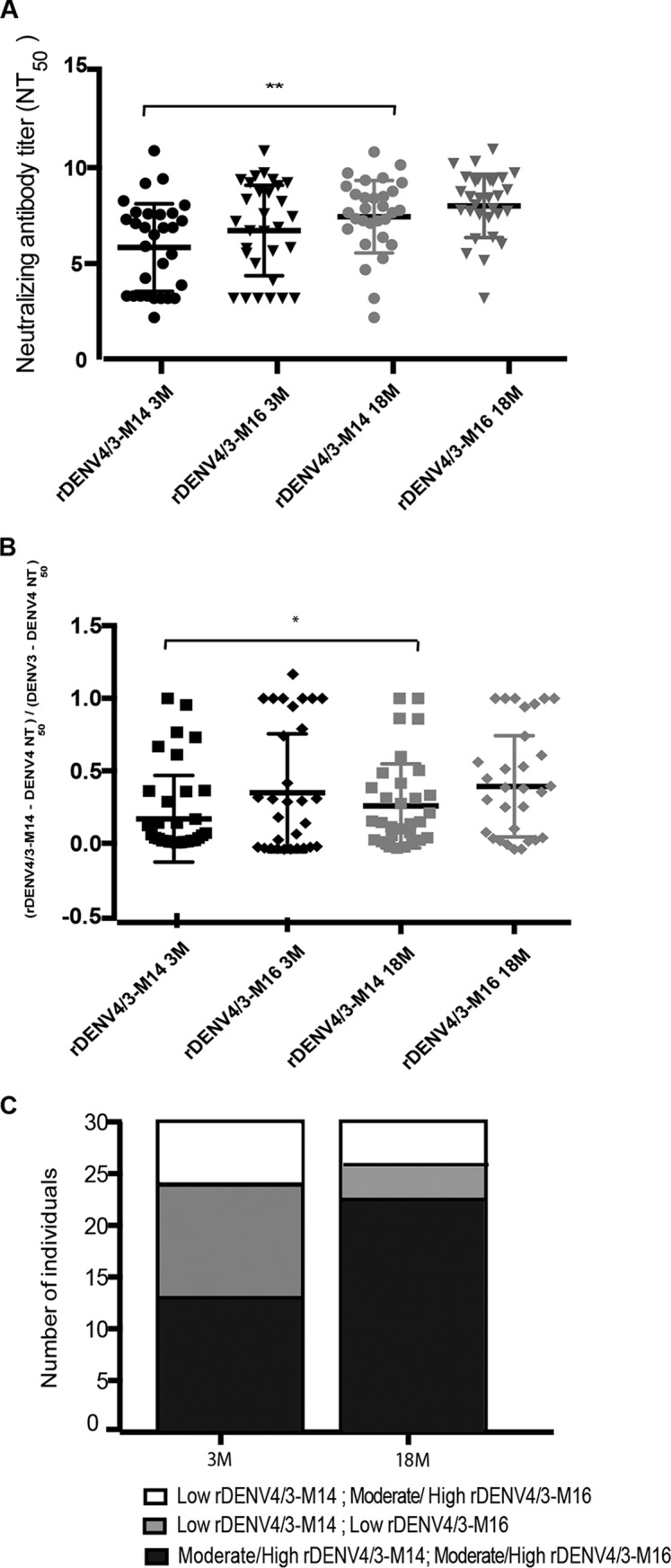
Neutralizing titers and proportion of the DENV3 response attributable to the 5J7 amino acid residues contained in the rDENV4/3-M14 and rDEV4/3-M16 viruses 3 and 18 months post-primary DENV3 infection. (A) Neutralizing antibody titers to rDENV4/3-M14 and to the chimeric virus with a larger footprint of the 5J7 epitope (rDENV4/3-M16) in primary DENV3 plasma 3 and 18 months postinfection (*n* = 30). (B) Proportion of the DENV3 neutralizing response attributable to the 5J7 amino acid residues transplanted into the DENV4 backbone in the rDENV4/3-M14 and rDENV4/3-M16 viruses 3 and 18 months post-primary DENV3 infection (*n =* 30). (C) Representation of the number of individuals who strongly recognize the rDENV4/3-M14 and rDENV4/3-M16 viruses or individuals who are refractory to rDENV4/3-M14 virus and only recognize the rDENV4/3-M16 virus at 3 and 18 months postinfection. The criteria for “low rDENV4/3-M14” or “low rDENV4/3-M16” was 0 to 10% of the DENV3 neutralizing response due to the 5J7 epitope, whereas “moderate/high rDENV4/3-M14” or “moderate/high rDENV4/3-M16” was defined as a proportion higher than 10%. The NT_50_ and the proportion of the 5J7 response were compared by two-way ANOVA (*n =* 30), where * represents *P* < 0.05 and ** represents *P* < 0.01.

To test whether some individuals were refractory to the rDENV4/3-M14 virus due to the inclusion of the 5J7 epitope transplant with contact sites on 2 DENV3 E monomers, as opposed to 3 monomers (9), a second chimeric virus was generated (Widman et al., unpublished). This chimeric virus, designated here rDENV4/3-M16, contains the expanded 5J7 epitope transplant to encompass all three monomers (Fig. 1B). Plasma samples from all subjects were tested against the new rDENV4/3-M16 virus to determine whether the entire structurally defined footprint of the 5J7 epitope confers a gain of neutralization compared to the rDENV4/3-M14 virus (Fig. 6; see Fig. S4 in the supplemental material). The neutralizing antibody titers due to rDENV4/3-M16 were slightly higher than those of rDENV4/3-M14 at 3 and 18 months post-infection, but not significantly so (Fig. 6A). The expansion of the quaternary 5J7 epitope in the rDENV4/3-M16 virus in individuals who presented a moderate or high degree of recognition of the rDENV4/3-M14 virus did not result in a significant increase of the proportion of the DENV3 response attributable to the 5J7 epitope at either time point analyzed (Fig. 6B). However, in individuals who displayed low neutralization of the rDENV4/3-M14 virus at 3 months (*n =* 17), a gain of neutralization to the rDENV4/3-M16 virus was observed in 6 individuals at 3 months post-infection (Fig. 6C). At 18 months, 4 individuals who displayed low neutralization of rDENV4/3-M14 (*n =* 8) gained neutralization to the rDENV4/3-M16 virus (Fig. 6C). However, the fact that a number of individuals still did not recognize the 5J7 epitope at 3 months (*n =* 11) and 18 months (*n =* 4) even in an expanded structure suggests that other DENV3 type-specific neutralizing epitopes contribute to the DENV3-specific epitope repertoire.

10.1128/mBio.01205-17.4 FIG S4  Neutralizing antibody titers to rDENV4/3-M14 and rDENV4/3-M16 and respective proportion analysis at 3 and 18 months postinfection. (A to L) Individuals with higher recognition of the rDENV4/3-M16 compared to the rDENV4/3-M14 virus at 3 and/or 18 months post-infection. (M to ZD) Individuals with similar recognition of the rDENV4/3-M16 compared to the rDENV4/3-M14 virus at 3 and/or 18 months post-infection. Download FIG S4, PDF file, 0.5 MB. Copyright © 2017 Andrade et al. 2017 Andrade et al. This content is distributed under the terms of the Creative Commons Attribution 4.0 International license.

## DISCUSSION

Defining the antibody repertoire in natural DENV infections and vaccination is critical to understanding the quality of neutralizing antibody responses *in vivo*. Isolation of hMAbs from DENV-immune individuals is one approach, which has greatly advanced the field (7–10, 19, 20); however, the next critical step is studying human polyclonal sera from individuals living in regions where dengue is endemic to analyze the prevalence of specific epitopes of interest over time. Our findings on the prevalence and durability of a DENV3 type-specific epitope in a population of dengue endemicity up to 4 years after infection indicate that the 5J7 epitope is an important epitope within a larger DENV3 repertoire that requires further study. In addition, our study provides insights regarding the footprint of the epitope and demonstrates that chimeric viruses represent a powerful tool for mapping type-specific responses following natural infection at the population level.

The recognition of a quaternary DENV2 epitope transplant in a chimeric virus by polyclonal sera has been previously demonstrated in rhesus macaque DENV-immune sera and human DENV-immune sera from travelers and vaccinees (12). The successful transplantation of the amino acids residues contained within the 5J7 epitope into a DENV4 backbone provides an important tool for identification of antigenic sites specific to polyclonal neutralizing antibodies following natural DENV3 infection. A recent study demonstrated that the 5J7 MAb neutralizes the rDENV4/3-M16 virus more efficiently than rDENV4/3-M14 and that returned travelers’ sera partially tracked with the rDENV4/3-M14 and showed increased neutralizing antibody responses to the rDENV4/3-M16 virus (Widman et al., unpublished). Here, we show that large numbers of human plasma samples from a population living in a region where dengue is endemic validate the footprint of the 5J7 epitope.

Following a primary DENV infection, an increasingly type-specific response over time is described in sera of individuals living in areas of nonendemicity (21) and returned travelers who experienced DENV infection (7). We found that while the parental DENV3 titers did not change over time, the neutralizing response to the rDENV4/3-M14 virus increased from 3 to 18 months. This suggests that the DENV3 type-specific response recognizing the 5J7 epitope is enriched/selected months after infection. Supporting evidence that the proportion of the DENV3 neutralizing response directed to the 5J7 epitope increases over time is that antigenic cartography maps show convergence of the rDENV4/3 and parental DENV3 viruses from 3 to 18 months.

This increase in specificity could reflect the evolution and diversity of the antibody repertoire due to stochastic events in the germinal center and mutations during affinity maturation following infection. Memory B cells and long-lived plasma cells develop from naïve B cells and undergo affinity maturation during several months after infection (22, 23). The improved recognition of the 5J7 epitope at 18 months could suggest the presence of viral antigens in peripheral organs that may sustain affinity maturation of the epitope over time. However, contrary to Zika virus (24), West Nile virus (25), Japanese encephalitis virus (26), tick-borne encephalitis virus (27), and Ebola virus (28), to our knowledge no studies have detected DENV in peripheral sites months after infection. Therefore, the gain of neutralization to the 5J7 epitope from 6 to 18 months may not be attributed to ongoing affinity maturation months after infection triggered by persistence of DENV antigens.

The levels of cross-reactive antibodies are thought to decay over time in regions of nonendemicity (21). However, the trajectory of cross-reactive titers at 3, 6, 12, and 18 months post-infection revealed an increase in NT_50_ titers in a subset of plasma samples analyzed. This finding is consistent with a previous study by our group demonstrating a modest increase in the magnitude and cross-reactivity of the neutralizing antibody response following primary DENV infection in Nicaragua (16). The observation of “boosts” in neutralizing antibody titers suggests that reinfection with the homologous serotype or possibly low-level heterotypic infection may have occurred. These findings are consistent with another longitudinal study in Thailand, which also demonstrated an increase in cross-reactive neutralizing antibody titers over time (17). In the light of these findings, reexposure to DENV may be an important factor modulating the long-term antibody response. As such, it may also affect the serotype-specific epitope repertoire following DENV infection. For instance, reexposure to homotypic virus, consistent with the epidemiology of DENV in our cohort study in Nicaragua, where one serotype predominantly circulates for several years at a time, could help explain the increase in the proportion of the neutralizing antibody response to the 5J7 epitope over time. On the other hand, boosting of cross-reactive titers between 6 and 18 months could explain the loss of rDENV4/3 recognition at 18 months observed in a few individuals.

While only a small fraction (<3%) of the DENV-specific memory B cell population produces potent neutralizing antibodies (29), we showed that the 5J7 epitope is a substantial target of neutralizing antibodies in DENV3-immune plasma months and up to 4 years post-infection. As the 5J7 epitope transplant was expanded to include contact sites on 3 rather than 2 E monomers, the number of individuals who recognized the 5J7 epitope increased. Overall, as most individuals recognized the epitope either partially or fully, this provides evidence that the 5J7 epitope is an important component of the DENV3 type-specific neutralizing repertoire in human populations. Furthermore, these studies demonstrate how analysis of human populations can help define the footprint of an epitope of interest.

However, that the remaining primary DENV3 plasma samples did not gain neutralization to even the chimeric virus displaying a larger footprint of the 5J7 epitope suggests that neutralizing antibodies may target other DENV3 type-specific epitopes or an epitope overlapping with the 5J7 site. Furthermore, the abundant cross-reactive weakly neutralizing antibodies in polyclonal serum may compete with a minor subpopulation of potently neutralizing antibodies targeting these quaternary epitopes through steric interference. Our data provide compelling evidence that the DENV3 type-specific neutralizing antibody repertoire contains additional epitopes. In fact, multiple type-specific neutralizing epitopes exist for other DENV serotypes, as reported for DENV1 (10, 20) and DENV4 (30). In addition, in the context of a polyclonal response, binding of antibodies to one specific epitope may result in changes in the accessibility of a different epitope due to alternative conformations of the E protein (31). As previously shown in EDIII (32), variations in the E sequence across DENV3 strains may also affect recognition and neutralization of the 5J7 epitope. Similarly, a high degree of individual variation in antibody epitope repertoire has also been observed in sera of individuals naturally infected or vaccinated with yellow fever virus (33) and tick-borne encephalitis virus (34).

In summary, the differential recognition of the 5J7 epitope among individuals could suggest (i) variability of the antibody repertoire within the host, (ii) the critical need for additional amino acid residues spanning the three E protein monomers contained within the 5J7 epitope that were not transplanted into the original chimeric rDENV4/3-M14 virus or even a greater expansion of the epitope(s) (rDENV4/3-M16), and/or (iii) the existence of other epitopes in the DENV3 type-specific repertoire. This is the first large-scale study demonstrating that human plasma from a population living in an area where dengue is endemic can validate and help define the footprint of a quaternary epitope. Further, our results strongly suggest that 5J7 epitope is an important component of a larger DENV3 type-specific neutralizing epitope repertoire. Moreover, the use of chimeric viruses as a molecular tool to decipher the neutralizing response in a human population has a direct impact on guiding vaccine development and measurement of type-specific response following DENV infection.

## MATERIALS AND METHODS

### Ethics statement.

The dengue hospital-based study and the Pediatric Dengue Cohort Study were approved by the Institutional Review Boards of the University of California, Berkeley, and the Nicaraguan Ministry of Health. Parents or legal guardians of the subjects enrolled in these studies provided written informed consent, and participants 6 years of age and older provided assent.

### Study population. (i) Dengue hospital-based study.

Study enrollment took place in the Nicaraguan national pediatric reference hospital, Hospital Infantil Manual de Jesús Rivera (HIMJR). Children between 6 months and 14 years old suspected of dengue (<7 days of illness) were eligible to participate in the hospital study, as described previously (35). Laboratory-confirmed dengue cases were classified by severity using a computerized algorithm that compiled all clinical data meeting each criterion for dengue fever (DF), dengue hemorrhagic fever (DHF), or dengue shock syndrome (DSS), as detailed in the 1997 WHO guidelines (1, 35). Based on these guidelines, 20 individuals were classified as DF, while 10 experienced DHF. Plasma samples were collected in the acute (days 1 to 6) and convalescent (days 14 to 28) phases, as well as 3, 6, 12, and 18 months post-onset of illness.

### (ii) Pediatric Dengue Cohort Study.

The Nicaraguan Pediatric Dengue Cohort Study (PDCS; 2004 to present) is a community-based prospective study of approximately 3,500 children 2 to 14 years of age in Managua, Nicaragua (36). Healthy annual blood samples collected from 10 participants 1, 2, 3, and 4 years after experiencing a primary DENV3 infection were used.

### Laboratory tests.

DENV infection was confirmed by RT-PCR for detection of viral RNA (37, 38), isolation of DENV on C6/36 cells (37), and/or seroconversion by IgM enzyme-linked immunosorbent assay (ELISA) or a ≥4-fold increase in total antibody titer as measured by inhibition ELISA in paired acute- and convalescent-phase samples. In the hospital study, primary dengue cases were determined by inhibition ELISA (39, 40), where antibody titers of <2,560 in the convalescent-phase sample (day 14 to 28 post-onset of symptoms) define primary infection status (13). In the cohort study, a primary DENV infection was classified by seroconversion (a titer of <1:10 to >1:10 as determined by inhibition ELISA) in paired consecutive annual samples (36).

### Cells and viruses.

U937 cells expressing DC-SIGN (dendritic cell-specific intercellular adhesion molecule-3-grabbing nonintegrin), a known DENV attachment factor, were maintained as suspension cell cultures at 37°C with 5% CO_2_ in RPMI 1640 (Gibco) supplemented with 1% non-essential amino acids, 1% penicillin and streptomycin, and 5% fetal bovine serum (FBS; HyClone). *Aedes albopictus* C6/36 cells were grown at 32°C in 5% CO_2_ and were used for propagation of the Nicaraguan strains of DENV1 (N1265), DENV2 (N172), and DENV3 (N7236), parental strains of DENV3 (UNC 3001) and DENV4 (Haiti 74), and recombinant viruses rDENV4/3-M14 and rDENV4/3-M16. The rDENV4/3-M14 virus contains 29 amino acid residues within the 5J7 epitope, spanning two E protein monomers, whereas the rDENV4/3-M16 virus contains 36 amino acid residues from the 5J7 epitope spanning three E protein monomers (Widman et al., unpublished).

### DENV neutralization assay.

A flow cytometry-based neutralization assay was used to measure DENV-specific neutralizing antibodies, as previously described (41). Briefly, DENV-immune plasma at an initial dilution of 1:5 were serially diluted 3-fold 8 times in RPMI supplemented with 2% FBS. An amount of virus that infects 15% of the cells (previously determined by virus titration) was added to the plasma dilutions and incubated for 45 min at 37°C. After 24 h, the cells were centrifuged at 252 × *g* for 5 min and resuspended in 100 µl of RPMI medium. Next, cells were fixed in 4% paraformaldehyde, incubated for 10 min at room temperature, and centrifuged at 252 × *g* for 5 min. Then, cells were blocked in permeabilization buffer (0.1% saponin, 5% bovine serum albumin in 1× phosphate-buffered saline [PBS]) for 30 min at room temperature. Cells were subsequently incubated with anti-E MAb 4G2 conjugated to Alexa 488, diluted 1:1,000 in blocking buffer (0.5% bovine serum albumin and 0.02% sodium azide in 1× PBS) for 25 min at room temperature. Cells were washed and resuspended in PBS. The percentage of infected cells was determined using a Guava flow cytometer (EMD Millipore) by gating Alexa 488-positive cells. The plasma dilution that reduced viral infection by 50% (50% neutralizing antibody titer [NT_50_]) was calculated by a nonlinear, 4-parameter dose-response regression analysis with Prism software (GraphPad), which is expressed as the reciprocal serum dilution. To eliminate the effect of nonspecific cross-reactivity in human plasma, DENV-naïve titers (average of 10 DENV-naïve individuals enrolled in the PDCS) were subtracted from DENV-immune titers. Quality control criteria for the sigmoidal dose-response regression fit included an absolute sum of squares of <0.2 and a coefficient of determination (*R*^2^) of >0.9.

### Antigenic cartography map.

Antigenic maps were generated from NT_50_ against DENV3 UNC 3001, DENV4 Haiti 74, and rDENV4/3-M14 obtained from antisera collected at 3 and 18 months post-infection, as previously described (42). The measured antigenic distance, *D*_*ij*_, between virus *i* and antiserum *j* was estimated as *D*_*ij*_ = log_2_(*b*_*j*_) − log_2_(*N*_*ij*_), where *b*_*j*_ is the NT_50_ titer for the virus best neutralized by each antiserum *j*, and *N*_*ij*_ is the NT_50_ for virus *i* and antiserum *j*. Error function, *E = ∑ije*(*D*_*ij*_, *d*_*ij*_), was minimized (5,000 independent optimizations) to estimate coordinates for viruses and antisera that produced antigenic maps with the least disagreement between Euclidean distances, *d*_*ij*_, and measured antigenic distances, *D*_*ij*_. Error was defined as *e*(*D*_*ij*_, *d*_*ij*_) = (*D*_*ij*_ − *d*_*ij*_)^2^ when the NT_50_ was within the limit of detection of the assay and *e*(*D*_*ij*_, *d*_*ij*_) = (*D*_*ij*_ − 1 − *d*_*ij*_)^2^{1/[1 + *e*^−10(*dij* − 1 − *dij*)^]} for NT_50_ values below the assay limit of detection. The minimum error antigenic map is shown on a grid indicating antigenic distances as 2-fold dilutions, in any direction, on the antigenic map. The shift in virus position from the 3- to 18-month maps is shown with arrows.

### Statistical analysis.

Statistical analysis was conducted using Prism Graph Pad 5.0 (La Jolla, CA) and R (version 3.1.3). Two-way analysis of variance (ANOVA) was used to compare the NT_50_ values to rDENV4/3, DENV3 and DENV4 viruses at 3 and 18 months post-illness. Linear regression analysis with Pearson coefficient was used to evaluate the correlation between rDENV4/3 virus and DENV3 or DENV4 at 3 and 18 months post-illness. The paired *t* test was used for comparing the proportions of the DENV3 neutralizing antibody response attributable to the 5J7 epitope between samples collected 3 and 18 months postillness. A *P* value of <0.05 was accepted as statistically significant.
