# Supplementary figures and images for: Analysis of Individuals from a Dengue-Endemic Region Helps Define the Footprint and Repertoire of Antibodies Targeting Dengue Virus 3 Type-Specific Epitopes
Source: mBio. 2017 Sep 19;8(5):e01205-17. doi: 10.1128/mBio.01205-17 (PMC5605938; doi:10.1128/mBio.01205-17)

Supplemental Figure S1

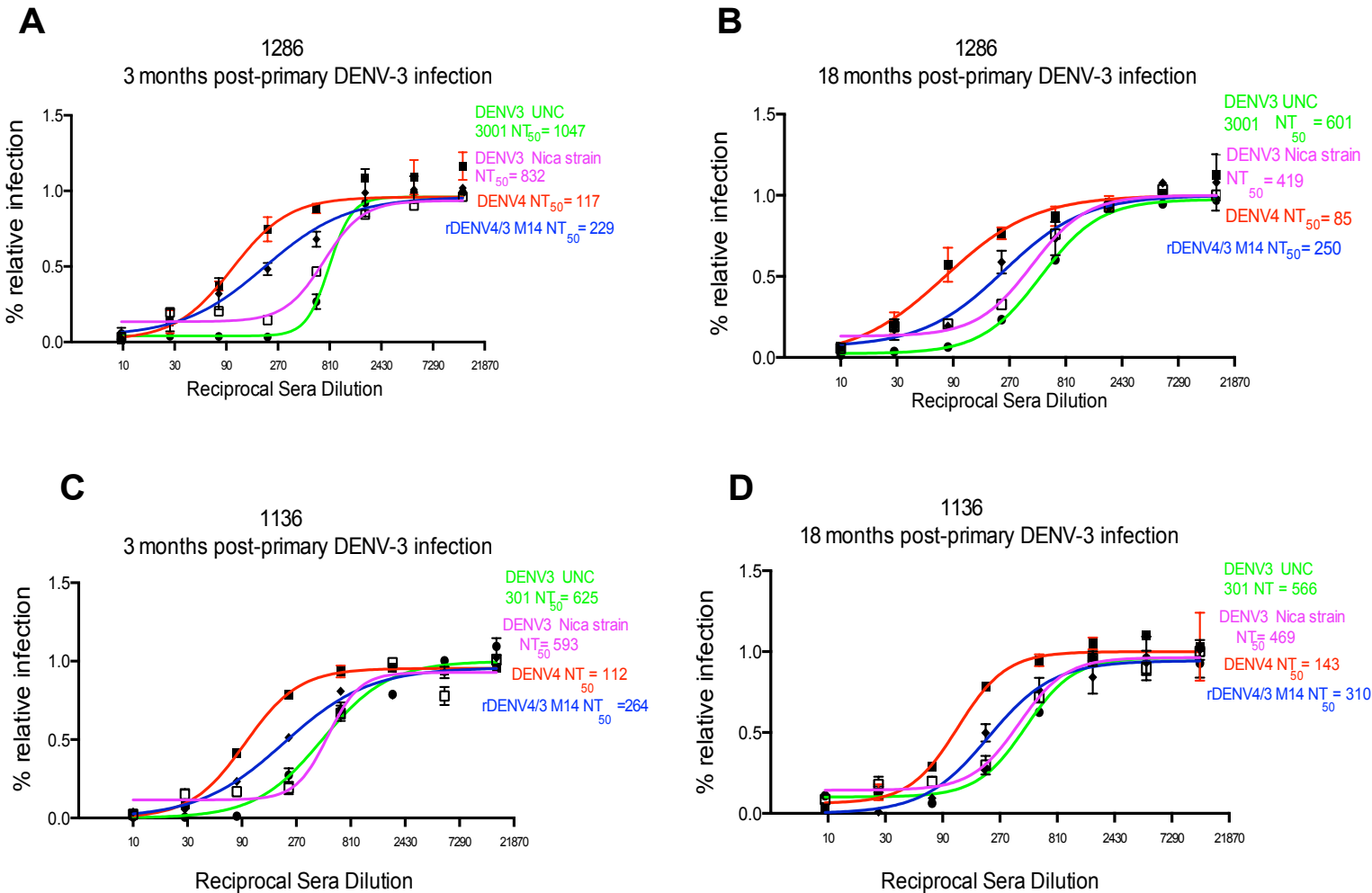

Supplement: FIG S1 [file mbo004173490sf1.pdf]

Supplemental Figure S2

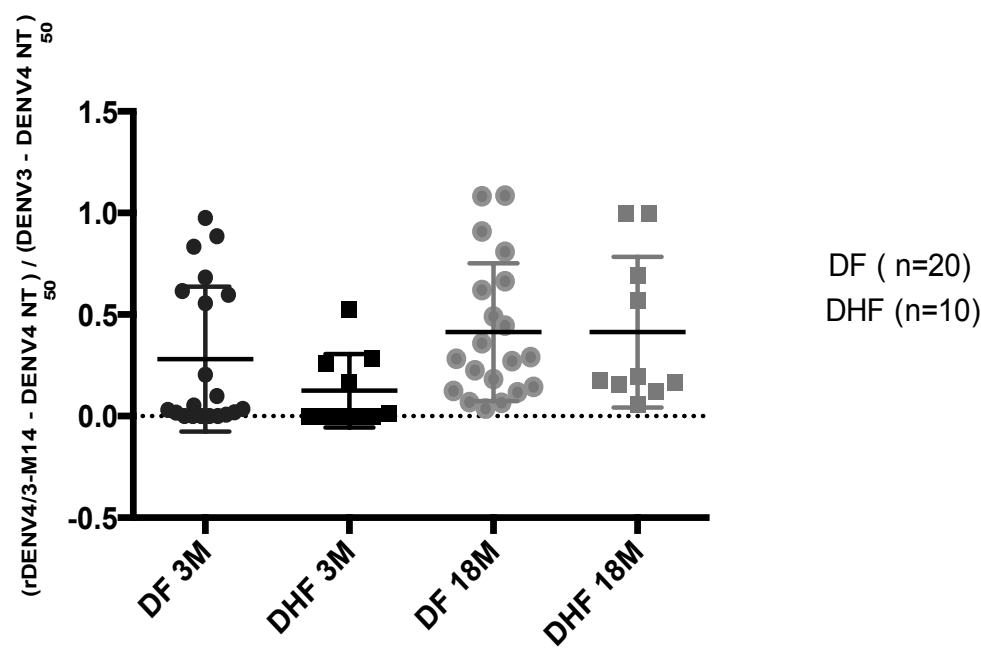

Supplement: FIG S2 [file mbo004173490sf2.pdf]

Supplemental Figure S3

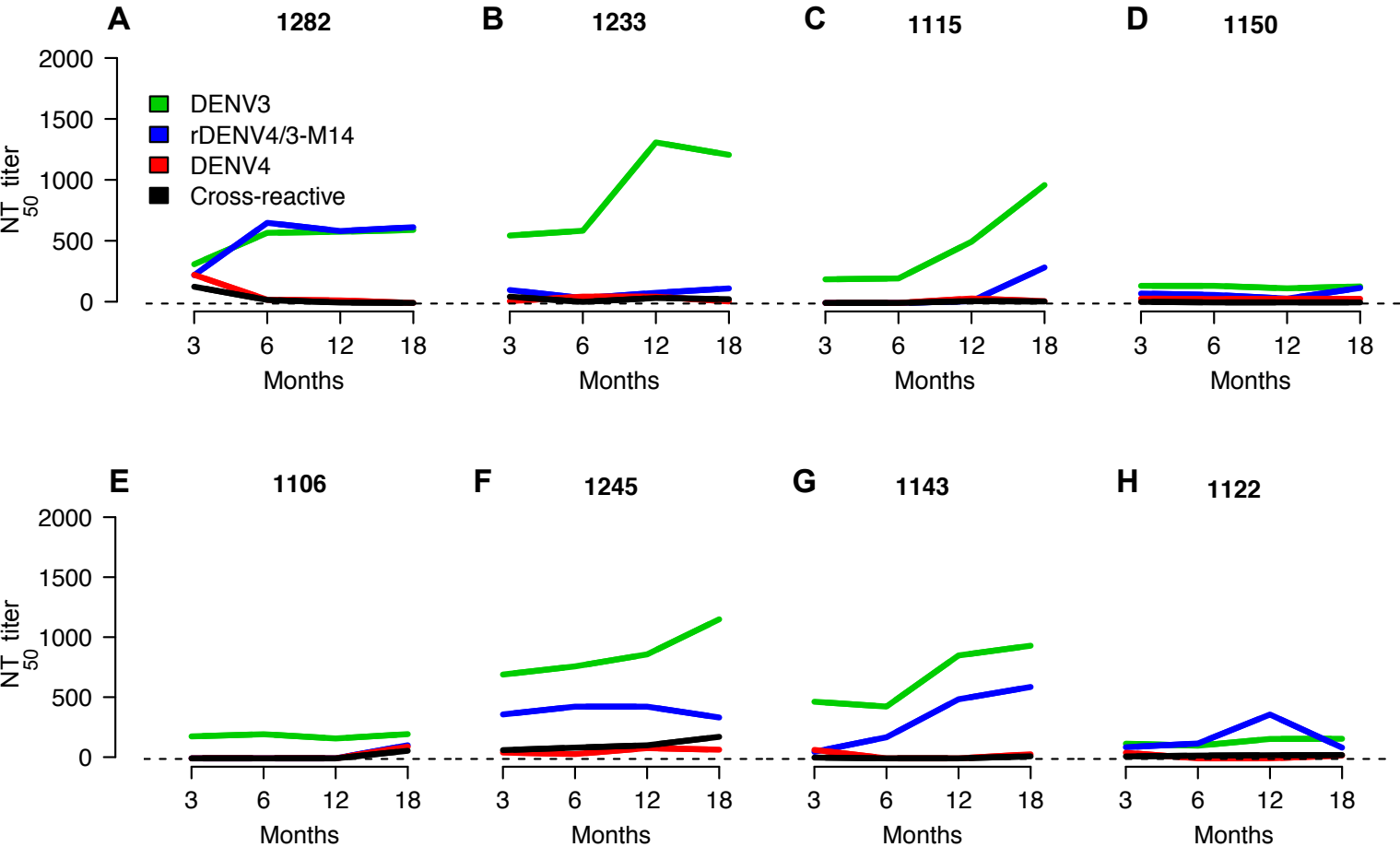

Supplement: FIG S3 [file mbo004173490sf3.pdf]

Supplemental Figure S4

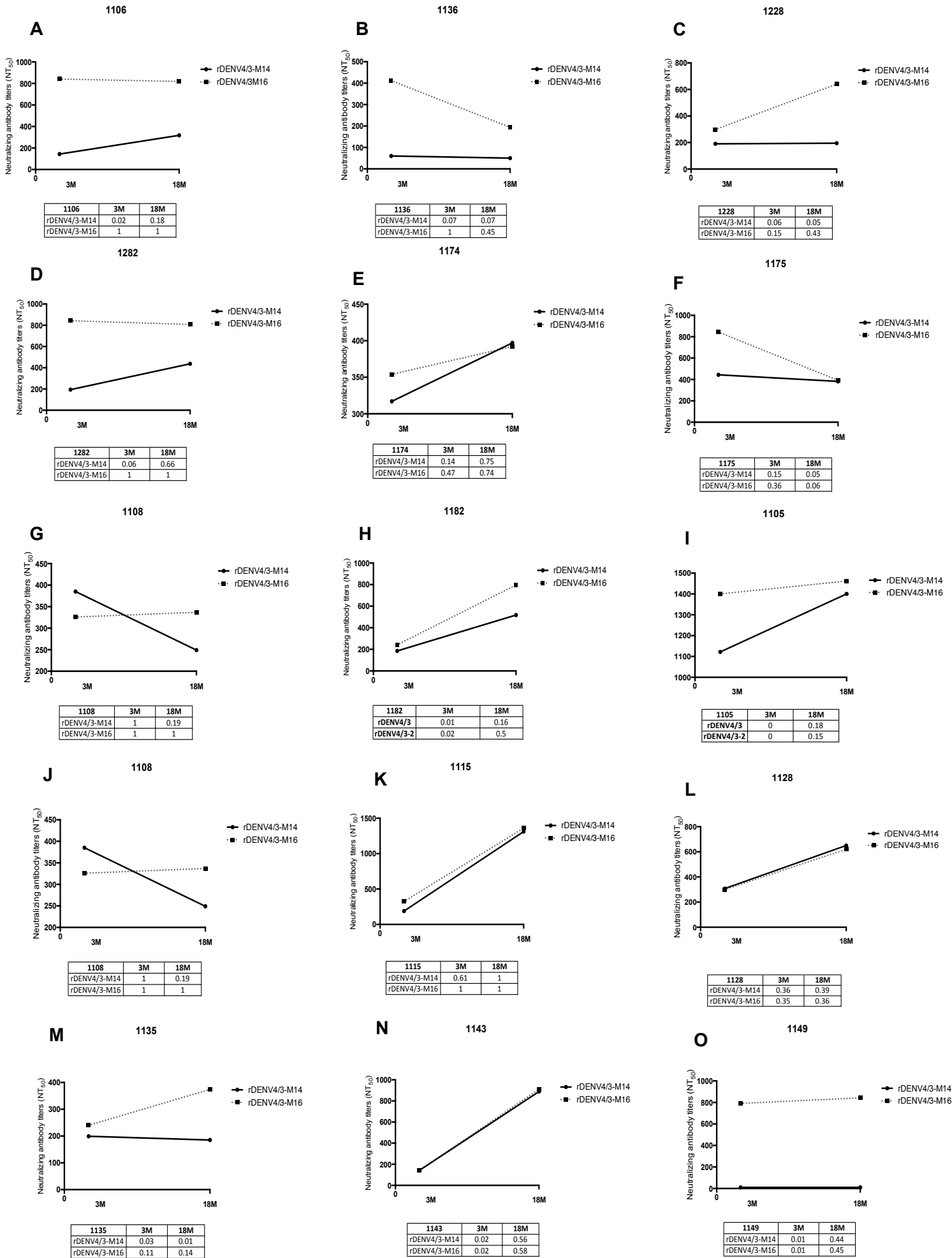

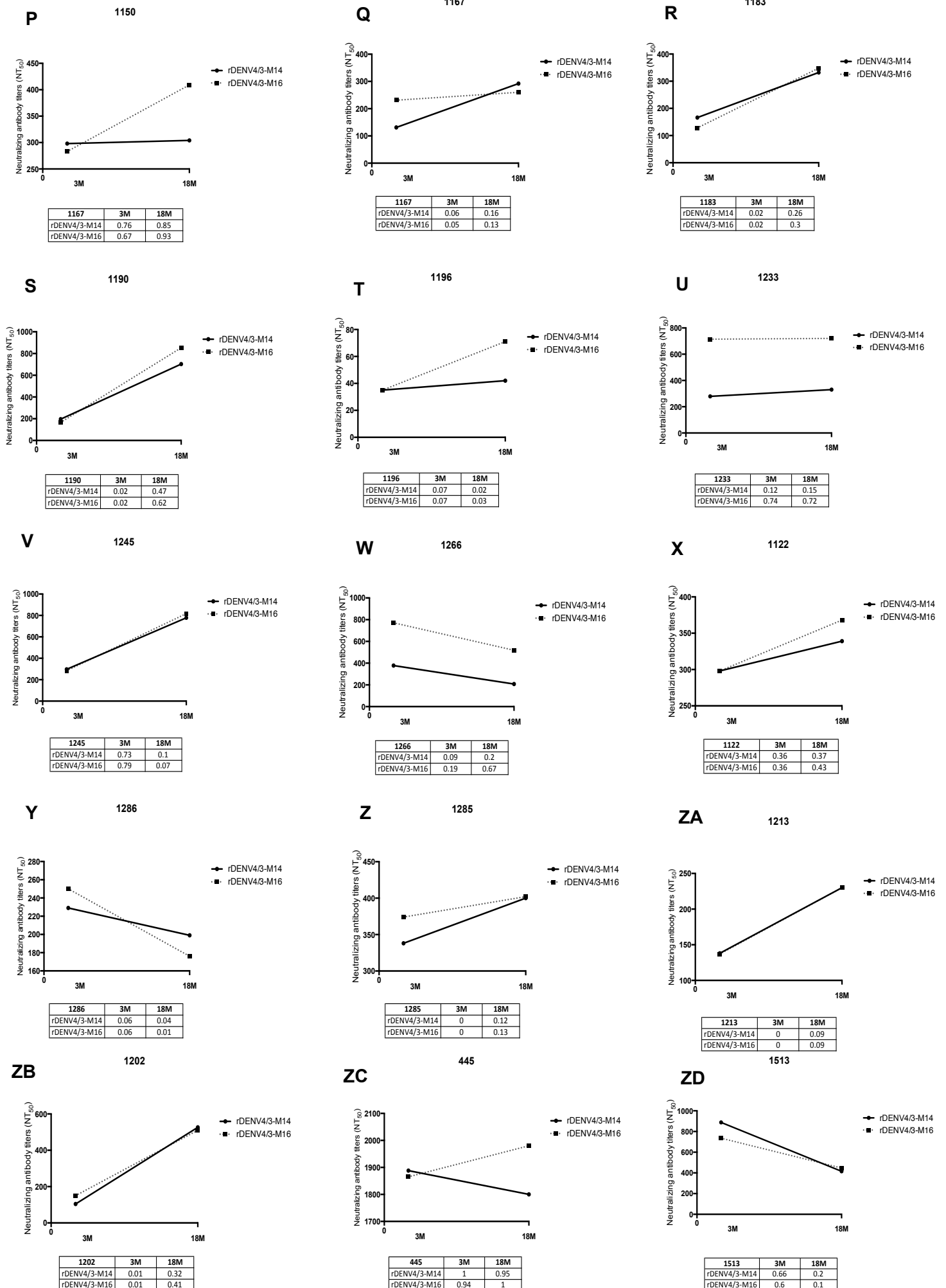

Supplement: FIG S4 [file mbo004173490sf4.pdf]
